# Galerkin finite element analysis for the augmentation in thermal transport of ternary-hybrid nanoparticles by engaging non-Fourier’s law

**DOI:** 10.1038/s41598-022-17424-4

**Published:** 2022-08-05

**Authors:** Muhammad Sohail, Umar Nazir, Essam R. El-Zahar, Choonkil Park, Wasim Jamshed, Kanit Mukdasai, Ahmed M. Galal

**Affiliations:** 1 Department of Mathematics, Khwaja Fareed University of Engineering & Information Technology, Rahim Yar Khan, 64200 Pakistan; 2 Department of Applied Mathematics and Statistics, Institute of Space Technology, P.O. Box 2750, Islamabad, 44000 Pakistan; 3 Department of Mathematics, King Saud University, Jeddah, Saudi Arabia; 4 Department of Basic Engineering Science, Faculty of Engineering, Menoufia University, Shebin El-Kom, 32511 Egypt; 5 Research Institute for Natural Sciences, Hanyang University, Seoul, 04763 Korea; 6 Capital Univeristy of Science and Technology (CUST), Islamabad, Pakistan; 7 Department of Mathematics, Faculty of Science, Khon Kaen University, Khon Kaen, 40002 Thailand; 8 Mechanical Engineering Department, College of Engineering, Prince Sattam Bin Abdulaziz University, Wadi Addawaser, Saudi Arabia; 9 Production Engineering and Mechanical Design Department, Faculty of Engineering, Mansoura University, P.O. 35516, Mansoura, Egypt

**Keywords:** Mathematics and computing, Nanoscience and technology

## Abstract

Boosting of thermal transportation is the demand of current era. Several techniques have been used to do so. One of an important way is the mixing of nanoparticles to boost thermal performance. Current investigation has been prepared to study the inclusion of tri hybrid nanoparticles in Prandtl fluid model past over a stretched heated sheet. Modelling of consider problem has been done due to consideration of movement in flow in Cartesian coordinates which results coupled partial differential equation system thermal transport in presented by considering generalized heat flux model and heat generation/absorption. The derived coupled complex partial differential equations (PDEs) system is simplified by engaging boundary layer theory. Such developed model is used in coolants regarding automobiles, dynamics in fuel and production of solar energy, fuel cells, optical chemical sensors, automotive parts, dental products, cancer therapy, electrical insulators and dental products. Handling of complex PDEs for the solution is a challenging task. Due to complexity in computational work these PDEs have been transformed into ordinary differential equations (ODEs) after applying similarity transformation afterwards converted ODEs have been approximated via finite element algorithm coded in MAPLE 18.0 symbolic computational package. Comparative study has been presented for the validity of code and authenticity of obtained result. It is observed that fluid velocity for tri-hybrid nanoparticles is higher than fluidic motion for pure fluid, nanofluid and hybrid nanomaterial.

## Introduction

Energy transport is an essential element of current era and hot topic of research area due to its wider applications in industry and different energy systems. Thermal transportation can be boosted by mixing the nanoparticles in the base fluid mixture. Several empirical relations of materials have been proposed due to materials characteristics. Researchers pay their attention particularly while modelling this model in different situation under different effects. Ternary hybrid nanomaterial is applicable in engineering process, cancer therapy, hair care products, electrical insulators, green tires, dental products, fuel cells, solar cells, optical chemical sensors, bio-sensors and automotive parts. For instance, Soomro et al.^1^ elaborated the involvement of nanoparticles passive control on convective thermal transmission in Prandtl past over an elongated sheet. They used boundary layer theory while deriving the considered flow presenting equations for simplification. After utilizing the similarity transformation resulting converted equations have been treated numerically via finite difference procedure. They have presented the streamlines against stretching parameter. Moreover, authenticity of scheme and obtained solution is shown by computing the heat transportation rate and obtained results are compared with the available open published data. Alsaedi et al.^2^ studied peristaltic transport phenomenon for MHD Prandtl model in a flexible symmetric channel under low Reynolds number principle. Perturbation approach was utilized to handle the derived modelled equations. Several important physical effects have been explored and explained by plotting graphs against numerous significant emerging parameters. They monitored the retardation in temperature field for Biot number and depreciation in velocity against Hartman parameter. Numerical approach has been used by Abbasi et al.^3^ to studied thermal transportation and mass transmission in Prandtl liquid flowing in an inclined channel obeying peristaltic phenomenon. They found the parabolic pattern of velocity field in the channel. Akbar et al.^4^ studied thermal transportation in Prandtl model in asymmetric channel using numerical and analytical tool. They derived the flow presenting expressions via low Reynolds number theory and long wavelength principle. They found an excellent settlement between the numerical and analytical solutions. They found the increment in pressure distribution against fluid parameter and channel’s amplitude. Bilal et al.^5^ used numerical approach to investigate mass and thermal transportation in electrically conducting Prandtl nanofluid flow. They analysed the impact of numerous parameters on mass and heat transportation rates through bar diagrams. Furthermore, they presented the comparative study. They recorded the augmentation in temperature and velocity fields against fluid parameter. Hayat et al.^6^ modelled the Prandtl liquid by engaging the generalized definitions for mass and heat fluxes past over a stretched surface in Cartesian coordinates. They engaged boundary layer theory to derive the flow equations. Afterwards, transformed modelled equations have been handled analytically via optimal homotopic approach. They analysed the retardation in concentration profile against Schmidt number and ratio parameter and depreciation in thermal field was observed for Prandtl number. Rajesh and Gowd^7^ studied peristaltic phenomena for Prandtl model by considering radiation. They used perturbation method for the solution. They computed the pressure and volume flow rate against numerous parameters and obtained results are displayed through graphs. They noticed the augmentation in pressure gradient against fluid parameter.

Due to enormous applications, study of heat and mass transportation has got remarkable consideration by the physicist, engineers and mathematicians. These mechanisms have been widely occurring in different processes in industry. These phenomena have been extensively reported by several researchers by considering different materials. For instance, Naseem et al.^8^ computed the analytic solution for third grade viscoelastic material with heat and mass transportation past over a Riga plate under generalized theories for mass and heat fluxes. They used boundary layer analysis for the derivation of flow presenting boundary value problem. Homotopic principle under basis function concept was utilized for the solution. They plotted the total error at 20th order of approximation. They recorded the enhancement in velocity field against Reynolds number and depreciation in concentration against Schmidt number. Rashidi et al.^9^ presented theoretical investigation on Burgers nanofluid model past over a stretchable surface under inclined magnetic field and mixed convection. They plotted the streamlines behaviour for Newtonian and Burgers model. Furthermore, validation of obtained solution is shown with the help of comparative study. They monitored the retardation in velocity against magnetic parameter and recorded the enhancement in fluid’s temperature and concentration against thermophoresis parameter. Polymeric bio-convective flow of Carreau liquid was examined by Prasad et al.^10^ numerically. They assumed the variable thickness upon which flow is produced. They considered slip boundary conditions for their developed problem. They recorded the decline in temperature, concentration and motile microorganism profile against escalating values of Weissenberg number. Also, they noticed the depreciation in heat transfer coefficient for different values of indexed number. Shehzad et al.^11^ examined the contribution of chemical reaction on Casson model. Srinivas and Kothandapani^12^ examined the MHD flow in compliant walls with peristaltic phenomenon and presented the perturbation solution. Ali et al.^13^ investigated enhancement in thermal energy using approach of hybrid nanofluid in the presence of viscous dissipation. Ahmed et al.^14^ used numerical approach to find the numerical consequences of heat transfer in the occurrence of nanoparticles in heated channel comprising variable viscosity. Gopal et al.^15^ discussed thermal features of nanofluid in mass diffusion and thermal energy involving chemical reaction and viscous dissipation under the magnetic field. Oke et al.^16^ studied thermal aspects based on nanoparticles in the presence heat source and coriolis force in the presence water. Saleem et al.^17^ studied the thermal aspects regarding nanoparticles and exergy in heated wavy channel. Few important contributions are covered in^18–31^.

**Figure 1:**
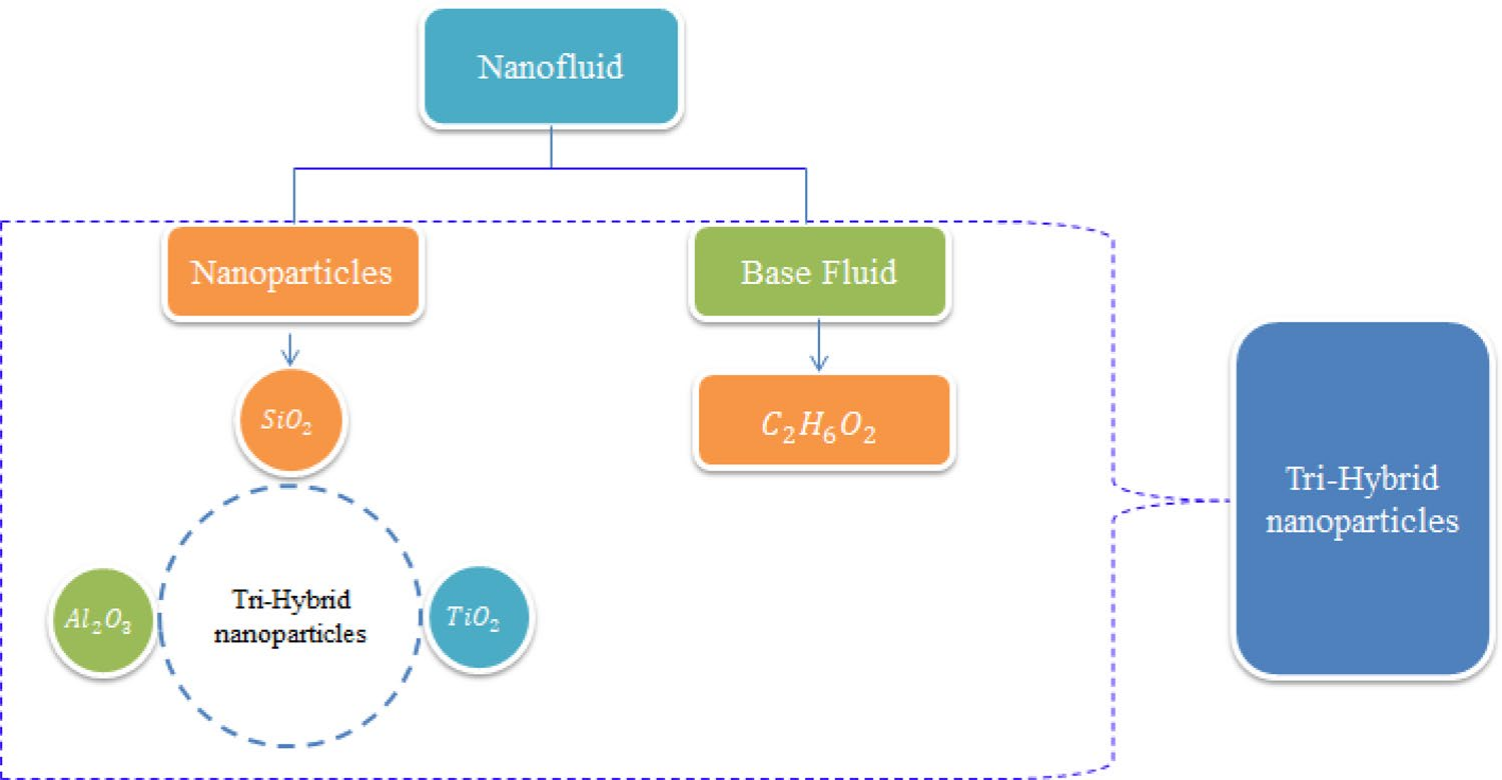
Preparation scheme of tri-hybrid nanoparticles.

The aim of this exploration is to mix tri hybrid nanoparticles in Prandtl fluid model to enhance thermal transportation. Available studies witness that so far no one attempted this study. This exploration will be used as a founding tool for the researchers to further explore different features and analyse numerous outcomes. This research has applications in numerous energies systems and industry. This report is organized as: literature survey is included in “Introduction” section, section covers the modelling of tri hybrid mixtures of nanoparticles for Prandtl model, an effective solution scheme is elaborated in “Mesh-free analysis and numerical approach” section with convergence criteria, outcomes of results with physical interpretation is listed in “Graphical discussion and outcomes” section and remarks have been incorporated in “Prime findings and consequences” section. Approach relate to tri-hybrid nanoparticles is prepared by Fig. 1.

## Modelling of heat transfer phenomena

**Figure 2:**
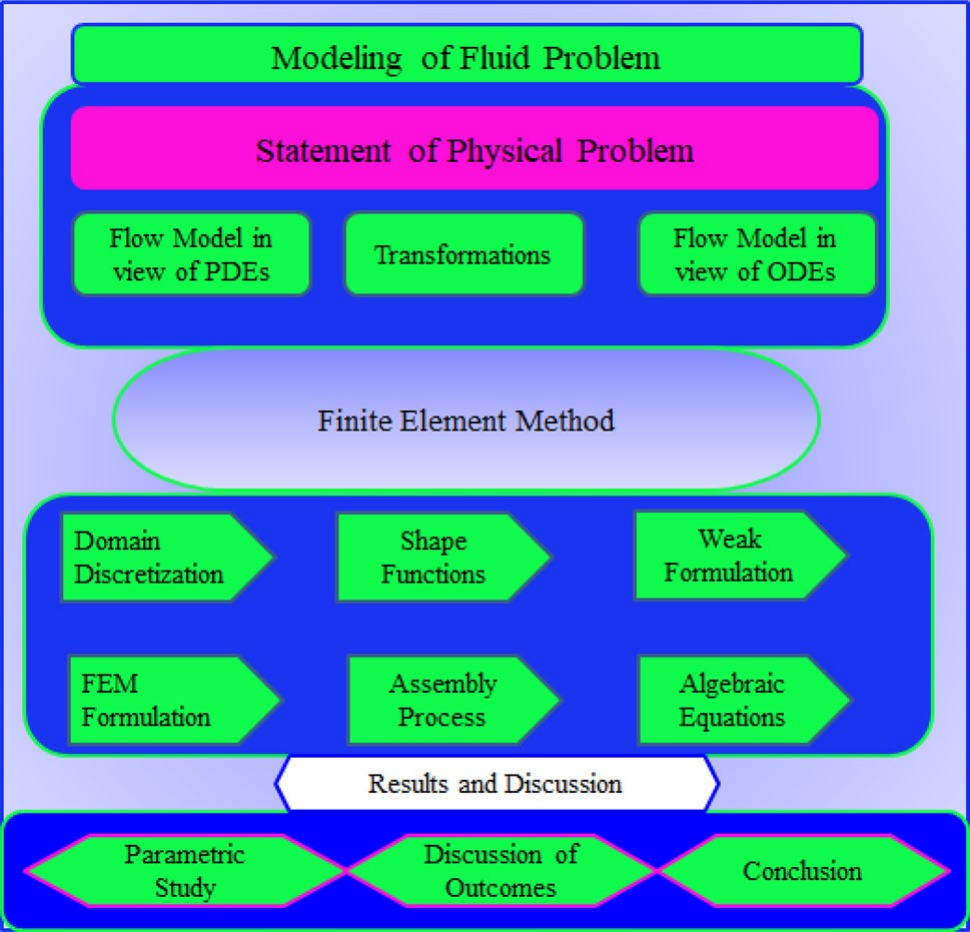
Work flow scheme of flow model.

**Figure 3:**
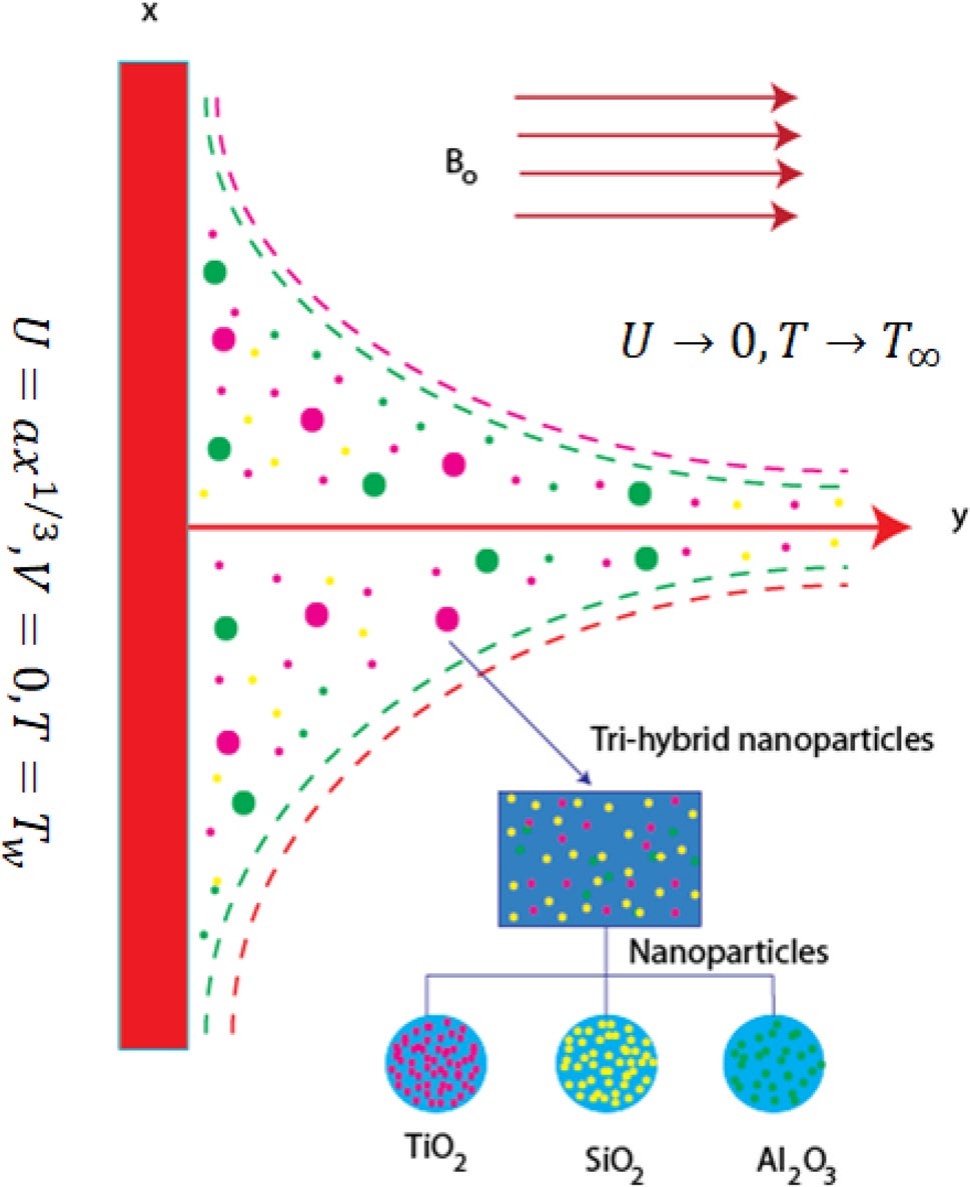
Physical model of developed model.

Motion of tri-hybrid nanoparticles in Prandtl fluid is observed past over a heated stretched surface. It is noticed that composition of three kind of nanoparticles (${Al}_{2}{O}_{3}, Ti{O}_{2}$ and $Si{O}_{2}$) in base fluid (ethylene glycol) is observed. The fluid runs due to stretching of the wall along horizontal direction of porous melting surface. The variable strength of magnetic field is considered along with rheology of Prandtl fluid. The complex modeling of non-Fourier’s within heat generation term is inserted in heat energy equation. BLA (boundary layer approximations) are used in basic laws of motion while these happenings provide set of PDEs. The work flow scheme of flow model is prepared by Fig. 2. The physical arrangement of current model is considered by Fig. 3.

Prandtl model^5^ is an important material whose constitutive relation is

1 $$\tilde{\varvec{\tau }} = \frac{{ASin^{ - 1} \left( {\frac{\partial U}{{\partial y}}\frac{\partial U}{{\partial y}}\frac{1}{C} + \frac{\partial V}{{\partial x}}\frac{\partial V}{{\partial x}}} \right)^{1/2} }}{{\left( {\frac{\partial U}{{\partial y}}\frac{\partial U}{{\partial y}} + \frac{\partial V}{{\partial x}}\frac{\partial V}{{\partial x}}} \right)^{1/2} }}\frac{\partial U}{{\partial y}}.$$

PDEs are developed using different physical effects and formulated PDEs^5,26–28^ are listed as

2 $$\frac{\partial U}{\partial x}+\frac{\partial V}{\partial y}=0,$$

3 $$\begin{aligned}U\frac{\partial U}{\partial x}+V\frac{\partial U}{\partial y}&=-\frac{{\sigma }_{Thnf}{B}_{0}^{2}{A}^{2}{x}^{-\frac{2}{3}}}{{\rho }_{Thnf}}U+{\nu }_{Thnf}\frac{A}{C}\left[\frac{{\partial }^{2}U}{\partial {y}^{2}}+\frac{1}{2{C}^{2}}{\left(\frac{\partial U}{\partial y}\right)}^{2}\frac{{\partial }^{2}U}{\partial {y}^{2}}\right]\\ &\quad+G{\beta }_{1}\left(T-{T}_{\infty }\right)-{\nu }_{Thnf}\frac{U}{{K}_{*}},\end{aligned}$$

4 $$\begin{aligned}U\frac{\partial T}{\partial x}+V\frac{\partial T}{\partial y}&=\frac{{K}_{Thnf}}{{\left(\rho {c}_{p}\right)}_{Thnf}}\frac{{\partial }^{2}T}{\partial {y}^{2}}{-\lambda }_{A}\left(\begin{array}{l}U\frac{\partial U}{\partial x}\frac{\partial T}{\partial x}+V\frac{\partial V}{\partial y} \frac{\partial T}{\partial x}+U\frac{\partial V}{\partial x}\frac{\partial T}{\partial y}+2UV\frac{{\partial }^{2}T}{\partial x\partial y}\\ {U}^{2}\frac{{\partial }^{2}T}{\partial {x}^{2}}+{V}^{2}\frac{{\partial }^{2}T}{\partial {y}^{2}}-\frac{Q}{{\left(\rho {C}_{p}\right)}_{Thnf}}\left(U\frac{\partial T}{\partial x}+V\frac{\partial T}{\partial y}\right)\end{array}\right)\\ &\quad+\frac{{K}_{Thnf}}{{(\rho {C}_{p})}_{Thnf}}\frac{{\partial }^{2}T}{{\partial }^{2}y}+Q\left(T-{T}_{\infty }\right).\end{aligned}$$

Using concept of no slip approach for development of BCs (boundary conditions) and BCs^29^ are

5 $$\left.\begin{array}{l}U=a{x}^\frac{1}{3}, \,\,V=0,\,\, T={T}_{w}\,\, at\,\, y=0\\ U\to 0,\,\, T\to {T}_{\infty }\,\, at\,\, y\to \infty .\end{array}\right\},$$

Transformations^29^ of current study are

6 $$\left.\begin{array}{l}U=a{x}^\frac{1}{3}f\left(\xi \right), V=-\sqrt{\frac{2a{\nu }_{f}}{3}}{x}^{-\frac{1}{3}}\left[f\left(\xi \right)-\frac{1}{2}\xi {f}^{{\prime}}\left(\xi \right)\right]\\ \theta \left(\xi \right)=\frac{T-{T}_{\infty }}{{T}_{w}-{T}_{\infty }}, \xi =\sqrt{\frac{2a}{3{\nu }_{f}}}{x}^{-\frac{1}{3}}y,\end{array}\right\}$$

Equations (2)–(5) are transformed in system of ODEs which are

7 $$\alpha f^{\prime\prime\prime} + \beta f^{{\prime\prime}{2}} f^{\prime\prime\prime} + \frac{{\nu_{Thnf} }}{{\nu_{f} }}\left[ {ff^{\prime\prime} - \frac{1}{2}f^{{\prime}{2}} } \right] - M^{2} \frac{{\sigma_{Thnf} }}{{\sigma_{f} }}f^{\prime} + \frac{{\nu_{Thnf} }}{{\nu_{f} }}\delta_{1} \theta - k_{1} f^{\prime} = 0,$$

8 $$\begin{aligned} &\theta^{\prime\prime} + \frac{{(\rho C_{p} )_{Thnf} k_{f} }}{{(\rho C_{p} )_{f} k_{Thnf} }}Prf\theta ^{\prime} - \frac{{k_{f} (\rho C_{p} )_{Thnf} }}{{k_{Thnf} (\rho C_{p} )_{f} }}Pr{\Omega }_{a} [ff^{\prime}\theta ^{\prime} + f^{2} \theta ^{\prime\prime} + H_{s} f\theta ^{\prime}]\\ &\quad + \frac{{k_{f} }}{{k_{Thnf} }}H_{s} Pr\theta = 0, \end{aligned}$$

9 $$\theta \left(0\right)=1, \,\,\theta \left(\infty \right)=0,\,\, f\left(0\right)=0,\,\, {f}^{{\prime}}\left(0\right)=1,\,\, {f}^{{\prime}}\left(\infty \right)=0.$$

Thermal properties related to density, viscosity, thermal and electrical conductivities for tri-hybrid nanoparticles^27,28^ are delivered as follows and numerical values are included in Table 1.

**Table 1:** Thermal properties related nanoparticles in EG^21,27^.

	$K$ (thermal conductivity)	$\sigma$ (electical conductivity)	$\rho$ (density)
${C}_{2}{H}_{6}{O}_{2}$	0.253	4.3 × 10^−5^	1113.5
$A{l}_{2}{O}_{3}$	32.9	5.96 × 10^7^	6310
$Ti{O}_{2}$	8.953	2.4 × 10^6^	4250
$Si{O}_{2}$	1.4013	3.5 × 10^6^	2270

10 $${\rho }_{Thnf}=\left(1-{\varphi }_{1}\right)\left\{\left(1-{\varphi }_{2}\right)\left[\left(1-{\varphi }_{3}\right){\rho }_{f}+{\varphi }_{3}{\rho }_{3}\right]+{\varphi }_{2}{\rho }_{2}\right\}+{\varphi }_{1}{\rho }_{1},$$

11 $$\frac{{\mu }_{f}}{{\left(1-{\varphi }_{3}\right)}^{2.5}{\left(1-{\varphi }_{2}\right)}^{2.5}{\left(1-{\varphi }_{1}\right)}^{2.5}}, \,\,\,\frac{{K}_{hnf}}{{K}_{nf}}=\frac{{K}_{2}+2{K}_{nf}-2{\varphi }_{1}\left({K}_{nf}-{K}_{2}\right)}{{K}_{2}+2{K}_{nf}+{\varphi }_{2}\left({K}_{nf}-{K}_{2}\right)},$$

12 $$\frac{{K}_{Thnf}}{{K}_{hnf}}=\frac{{K}_{1}+2{K}_{hnf}-2{\varphi }_{1}\left({K}_{hnf}-{K}_{1}\right)}{{K}_{1}+2{K}_{hnf}+{\varphi }_{1}\left({K}_{hnf}-{K}_{1}\right)},\,\,\, \frac{{K}_{nf}}{{K}_{f}}=\frac{{K}_{3}+2{K}_{f}-2{\varphi }_{3}\left({K}_{f}-{K}_{3}\right)}{{K}_{3}+2{K}_{f}+{\varphi }_{3}\left({K}_{f}-{K}_{3}\right)},$$

13 $$\frac{{\sigma }_{Tnf}}{{\sigma }_{hnf}}=\frac{{\sigma }_{1}\left(1+2{\varphi }_{1}\right)-{\varphi }_{hnf}\left(1-2{\varphi }_{1}\right)}{{\sigma }_{1}(1-{\varphi }_{1})+{\sigma }_{hnf}(1+{\varphi }_{1})}, \,\,\,\frac{{\sigma }_{hnf}}{{\sigma }_{nf}}=\frac{{\sigma }_{2}\left(1+2{\varphi }_{2}\right)+{\varphi }_{nf}\left(1-2{\varphi }_{2}\right)}{{\sigma }_{2}(1-{\varphi }_{2})+{\sigma }_{nf}(1+{\varphi }_{2})},$$

14 $$\frac{{\sigma }_{nf}}{{\sigma }_{f}}=\frac{{\sigma }_{3}\left(1+2{\varphi }_{3}\right)+{\varphi }_{f}\left(1-2{\varphi }_{3}\right)}{{\sigma }_{3}(1-{\varphi }_{3})+{\sigma }_{f}(1+{\varphi }_{3})}.$$

Here, Prandtl number, magnetic number, porosity number, bouncy number, heat generation number, time relaxation number fluid number and elastic number are expressed below.

$$\begin{aligned}&Pr\left(=\frac{{\mu }_{f}{\left(\rho {c}_{p}\right)}_{f}}{{k}_{f}}\right),\,\,\, {M}^{2}\left(=\frac{3{\sigma }_{Thnf}{B}_{0}^{2}{A}^{2}}{2a{\rho }_{f}}\right),\,\,\,{k}_{1} \left(=\frac{{\nu }_{f}}{{aK}_{*}}\right),\,\,\, {\delta }_{1}=\frac{G{\beta }_{1}{T}_{0}}{a{\nu }_{f}},\\ &\quad\alpha \left(=\frac{1}{C{A\mu }_{f}}\right),\,\,\, \beta \left(=\frac{{x}^{3}{a}^{3}}{2{C}^{2}{\nu }_{f}}\right),\,\,\, {\Omega }_{a}\left(={a\lambda }_{A}\right), \,\,\,{H}_{s}\left(=\frac{\mathrm{Q}}{{a\left({C}_{p}\right)}_{f}{\rho }_{f}}\right).\end{aligned}$$

Drag force in the presence of Prandtl liquid^21,27,28^ is

15 $$(Re)^{\frac{1}{2}} C_{f} = \frac{ - 1}{{(1 - \varphi_{3} )^{2.5} (1 - \varphi_{1} )^{2.5} (1 - \varphi_{2} )^{2.5} }}[\alpha f^{\prime\prime}(0) + \beta f^{\prime\prime}(0)^{3} ].$$

Temperature gradient (Nusselt number)^5,27,28^ in the presence of tri-hybrid nanoparticles is

16 $$Nu=\frac{x{Q}_{w}}{{k}_{f}\left(T-{T}_{\infty }\right)}, \,\,\,{Q}_{w}=-{k}_{Thnf}\frac{\partial T}{\partial y},$$

17 $$(Re)^{ - 1/2} Nu = \frac{{ - k_{Thnf} }}{{k_{f} }}\theta ^{\prime}(0).$$

The local Reynolds number is $Re\left(=\frac{a{x}^{2}}{{\nu }_{f}}\right).$

## Mesh-free analysis and numerical approach

ODEs along with BCs (boundary conditions) are simulated FEA (finite element approach)^29^. The following steps FEA are discussed below.

**Step I** Residuals of current ODEs along with BCs (boundary conditions) are modelled and weak procedures are made. The derivation of residuals is

18 $$\int_{{\eta_{e}}}^{{\eta_{{e + 1}}}} {w_{1} \left[ {F^{\prime } - H} \right]d\eta = 0,}$$

19 $$\mathop \int_{{\eta_{e}}}^{{\eta_{e + 1}}} w_{2} \left[ {\begin{array}{l} {\alpha H^{\prime\prime} + \beta H^{{\prime}{2}} H^{\prime\prime} + \frac{{\nu_{Thnf} }}{{\nu_{f} }}\left[ {fH^{\prime} - \frac{1}{2}f^{{\prime}{2}} } \right] - M^{2} \frac{{\sigma_{Thnf} }}{{\sigma_{f} }}H} \\ {\quad + \frac{{\nu_{Thnf} }}{{\nu_{f} }}\delta_{1} \theta - k_{1} H} \\ \end{array} } \right]d\eta = 0,$$

20 $$\mathop \int_{{\eta_{e}}}^{{\eta_{e+1}}} w_{3} \left[ {\begin{array}{l} {\theta ^{\prime\prime} + \frac{{(\rho C_{p} )_{Thnf} k_{f} }}{{(\rho C_{p} )_{f} k_{Thnf} }}Prf\theta ^{\prime} + \frac{{k_{f} (\rho C_{p} )_{Thnf} }}{{k_{Thnf} (\rho C_{p} )_{f} }}Pr{\Omega }_{a} [fH\theta ^{\prime} + f^{2} \theta ^{\prime\prime} + H_{s} f\theta ^{\prime}]} \\ \quad{ + \frac{{k_{f} }}{{k_{Thnf} }}H_{s} Pr\theta } \\ \end{array} } \right]d\eta = 0,$$

**Step II** GFES (Galerkin finite element scheme) is used to make weak forms in view of shape functions (linear). The shape functions are

21 $${\psi }_{i}={\left(-1\right)}^{i-1}\left(\frac{-\eta +{\eta }_{i-1}}{-{\eta }_{i}+{\eta }_{i+1}}\right),\,\,\, i=1, 2.$$

**Step III** Stiffness elements are formulated via assembly process and assembly process is done according to assembly procedure of FEA. Stiffness elements are formulated as

22 $$K_{{ij}}^{{11}} = \left( {\frac{{d\psi _{j} }}{{d\eta }}\psi _{i} } \right)d\eta ,K_{{ij}}^{{13}} = 0,K_{{ij}}^{{12}} = \smallint _{{\eta _{e} }}^{{\eta _{{e + 1}} }} \left( {\psi _{j} \psi _{i} } \right)d\eta , b_{i}^{1} = 0,$$

23 $${K}_{ij}^{22}=\int_{{\eta}_{e}}^{{\eta}_{e+1}}\left[\begin{array}{l}-\left(1+\beta \overline{{H }^{{\prime}}}\right)\frac{d{\psi }_{j}}{d\eta }\frac{d{\psi }_{i}}{d\eta }+\frac{{\nu }_{Thnf}}{{\nu }_{f}}\overline{f}\frac{d{\psi }_{j}}{d\eta }{\psi }_{i}-\frac{{\nu }_{Thnf}}{{\nu }_{f}}\frac{1}{2}\overline{H}{\psi }_{i}{\psi }_{j}\\ \quad -{M}^{2}\frac{{\sigma }_{Thnf}}{{\sigma }_{f}}{\psi }_{i}{\psi }_{j}-{k}_{1}{\psi }_{i}{\psi }_{j}\end{array}\right]d\eta ,$$

24 $${K}_{ij}^{23}=\int_{{\eta}_{e}}^{{\eta}_{e+1}}\left(\frac{{\nu }_{Thnf}}{{\nu }_{f}}{\delta }_{1}{\psi }_{i}{\psi }_{j}\right)d\eta , \,\,\,{K}_{ij}^{21}=0, \,\,{b}_{i}^{2}=0, \,\,{K}_{ij}^{31}=0, \,\,{K}_{ij}^{32}=0,$$

25 $${K}_{ij}^{33}=\int_{{\eta}_{e}}^{{\eta}_{e+1}}\left[\begin{array}{l}-\left(1+{\overline{f} }^{2}\right)\frac{d{\psi }_{j}}{d\eta }\frac{d{\psi }_{i}}{d\eta }+\frac{{k}_{f}{\left(\rho {C}_{p}\right)}_{Thnf}}{{k}_{Thnf}{\left(\rho {C}_{p}\right)}_{f}}Pr{\Omega }_{a}\overline{f }\overline{H}\frac{d{\psi }_{j}}{d\eta }{\psi }_{i}\\ \quad+\frac{{k}_{f}{\left(\rho {C}_{p}\right)}_{Thnf}}{{k}_{Thnf}{\left(\rho {C}_{p}\right)}_{f}}Pr{\Omega }_{a}{H}_{s}\overline{f}\frac{d{\psi }_{j}}{d\eta }{\psi }_{i}+\frac{{k}_{f}}{{k}_{Thnf}}{H}_{s}Pr{\psi }_{i}{\psi }_{j}\end{array}\right]d\eta ,\,\,\,{b}_{i}^{3}=0.$$

**Step IV** Assembly process provides algebraic system (nonlinear equations) whereas this system is linearized with help of Picard linearization;

**Step V** System related to algebraic equations is simulated via ${10}^{-5}$ (computational tolerance) using following stopping criteria;

26 $$\left|\frac{{\varkappa }_{i+1}-{\varkappa }_{i}}{{\varkappa }^{i}}\right|<{10}^{-5}.$$

**Step VI** Table 2 reveals investigation of mesh-free;

**Table 2:** Mesh free investigation of temperature and velocity at mid of each 300 elements.

Number of elements	$f^{\prime}\left( {\frac{{\xi_{max } }}{2}} \right)$	$\theta \left(\frac{{\xi }_{max}}{2}\right)$
30	0.02143708615	0.09785806596
60	0.01883222946	0.08692198852
90	0.01805787247	0.08365054537
120	0.01768647229	0.08207762647
150	0.01746847353	0.08115314712
180	0.01732510656	0.08054464967
210	0.01722365248	0.08011379539
240	0.01714807437	0.07979268999
270	0.01708959385	0.07954414533
300	0.01704300449	0.07934609949

**Step-VII:** Convergence analysis is established against 300 elements as shown in Table 2.

### Validation of problem

Present problem can be reduced in published work^29^ considering $\alpha =\beta ={k}_{1}={\delta }_{1}=0, {H}_{s}={\Omega }_{a}=0, {\varphi }_{1}=0.002, {\varphi }_{3}=0,{\varphi }_{2}=0.075.$ Validation simulations are recorded in Table 3. It is observed that good agreements are noticed among published study and present analysis.

**Table 3:** Validation of numerical consequence for Nusselt number and skin friction coefficient when $\alpha =\beta ={k}_{1}={\delta }_{1}=0, {H}_{s}={\Omega }_{a}=0, {\varphi }_{1}=0.002, {\varphi }_{3}=0,{\varphi }_{2}=0.075.$

Nazir et al.^29^		Present results	
${-\left(Re\right)}^{1/2}{C}_{f}$	${-\left(Re\right)}^{-1/2}Nu$	${-\left(Re\right)}^{1/2}{C}_{f}$	${-\left(Re\right)}^{-1/2}Nu$
0.9366353488	0.07196446469	0.93658959083	0.07119370518

## Graphical discussion and outcomes

Developed model in the presence of non-Fourier’s law along with heat generation is modelled. A vertical 2D porous surface is taken out to measure results of velocity and heat energy against various parameters. It is noticed tri-hybrid nanoparticles is considered as composite among three kinds of nanoparticles (${Al}_{2}{O}_{3}, Ti{O}_{2}$ and $Si{O}_{2}$) and hybrid nanoparticles is composition among two kind of nanoparticles ($Ti{O}_{2}$ and $Si{O}_{2}$) while base fluid is taken as ethylene glycol (EG)^30^. A FEA (finite element approach) is used to simulate graphical as well as numerical results. The main discussion related outcomes of flow and heat energy is discussed below.

### Comparative motion among nanoparticles via various parameters

**Figure 4:**
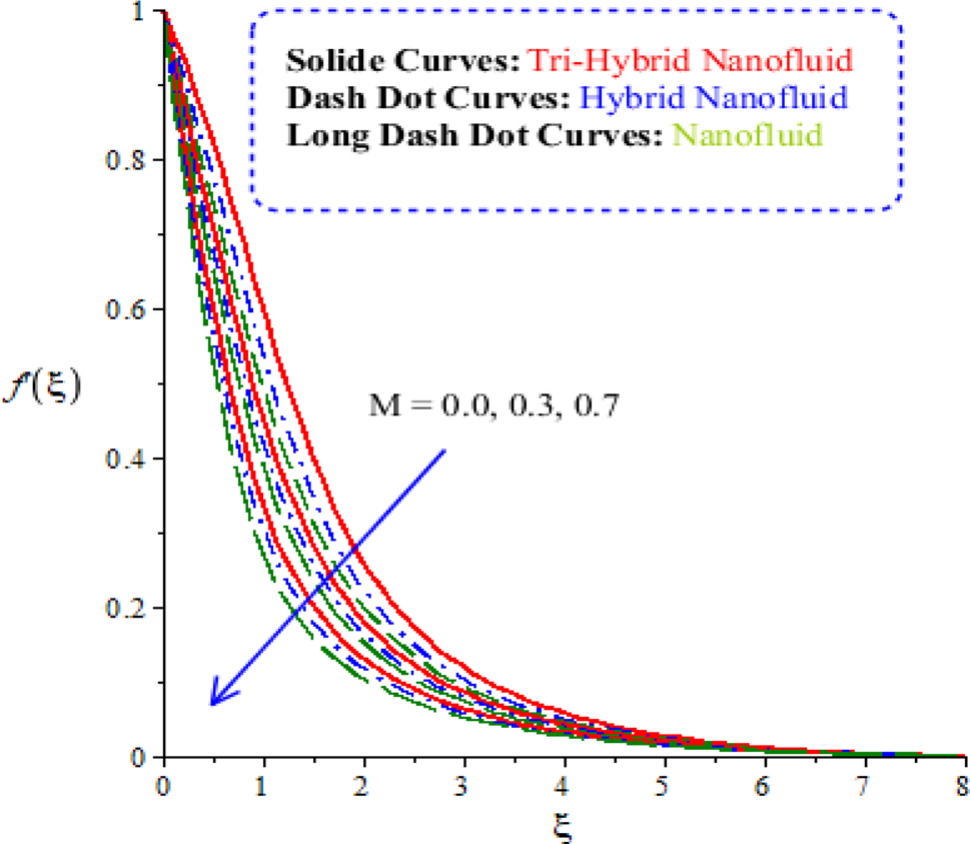
Comparative variation of velocity curves among nanoparticles versus $M.$

**Figure 5:**
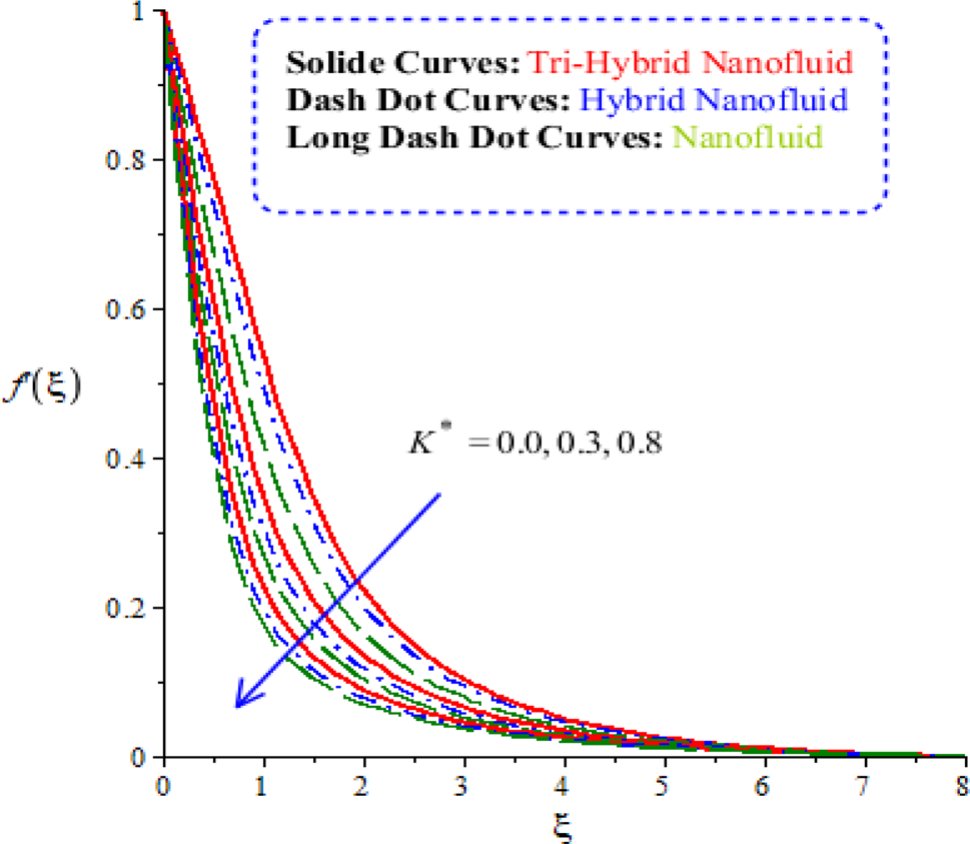
Comparative variation of velocity curves among nanoparticles versus ${K}^{*}.$

**Figure 6:**
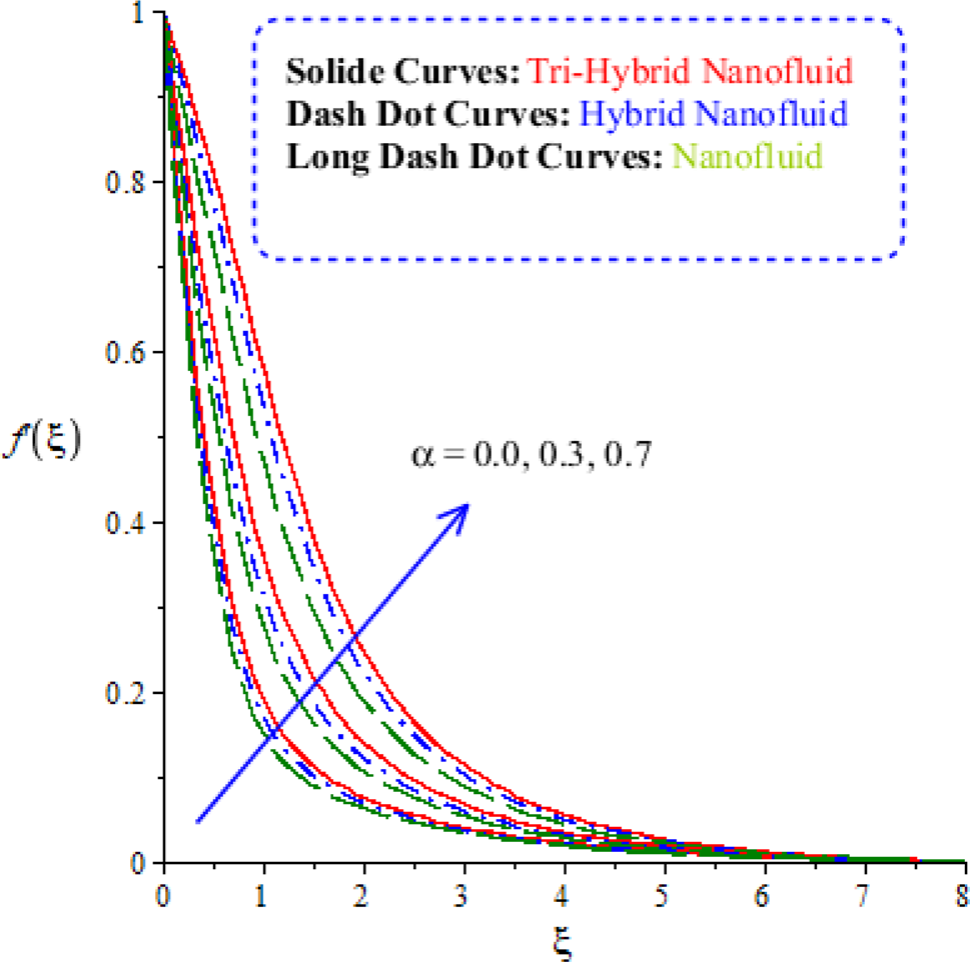
Comparative variation of velocity curves among nanoparticles versus $\alpha .$

**Figure 7:**
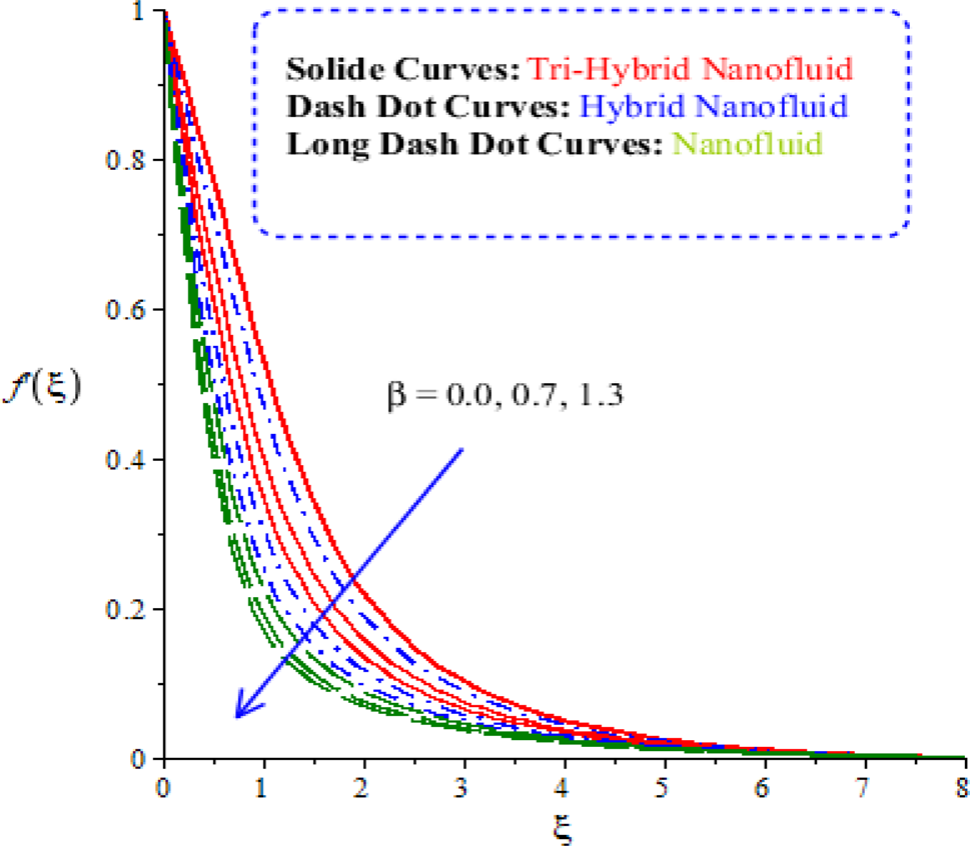
Comparative variation of velocity curves among nanoparticles versus $\beta .$

**Figure 8:**
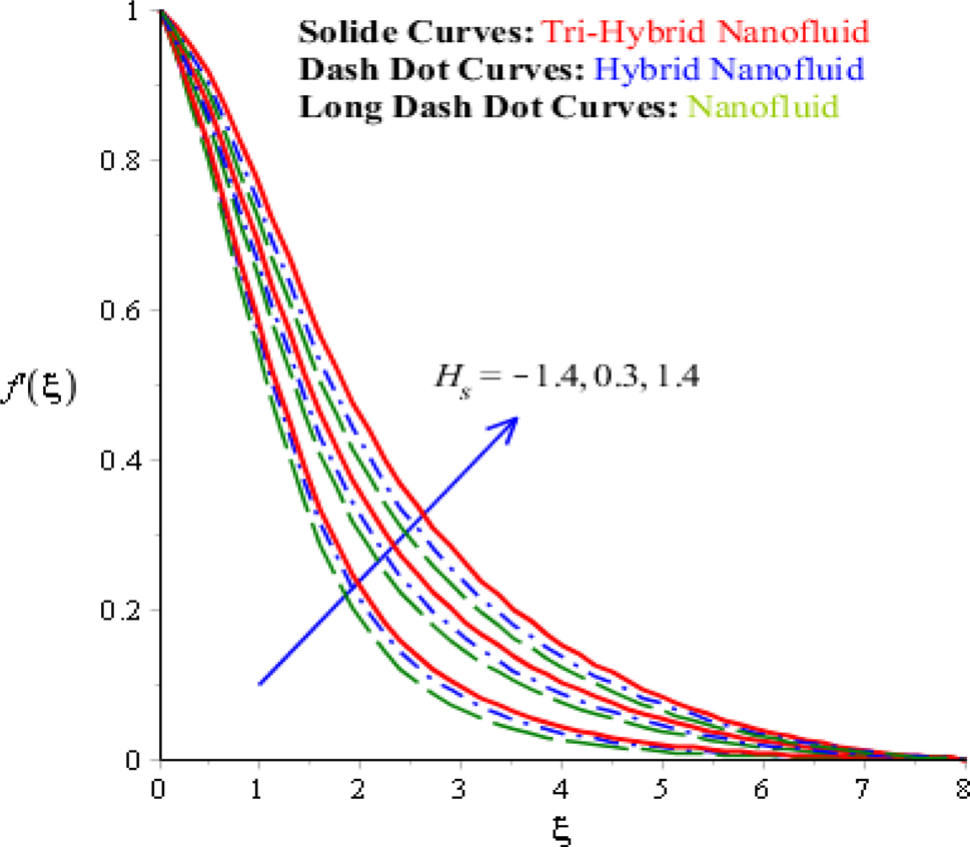
Comparative variation of velocity curves among nanoparticles versus ${H}_{s}.$

Characterizations of magnetic number, porosity parameter, and elastic parameter and Prandtl number are verified on the flow behaviour and heat energy among various nanoparticles. Related outcomes and comparative analysis versus temperature and velocity curves are captured by Figs. 4, 5, 6 and 7. It is noticed that dot curves related to graphs are identified by tri-hybrid nanoparticles and dash dot curves are represented the impact of hybrid nanoparticles while long dash dot curves are plotted for the role of nanoparticles. A comparative analysis among tri-hybrid nanoparticles, hybrid nanoparticles and nanoparticles in base fluid is conducted on flow variation against change in magnetic field (see Fig. 4). Velocity of nanoparticles is reduced when magnitude of magnetic number is enhanced. The reduction in the motion of hybrid nanoparticles, tri hybrid nanoparticles and nanoparticles are occurred due applied magnetic field. The direction of applied magnetic field is considered as normal during flow of nanoparticles. Therefore, reduction during flow of nanoparticles is created. Other reason is happened due to Lorentz force. Here, Lorentz force is appeared as a negative force in dimensionless momentum equations. So, this negative Lorentz force makes reduction in motion of hybrid nanoparticles. Moreover, motion of tri-hybrid nanoparticles is higher than motion created by hybrid nanoparticles and nanoparticles. Physically, Lorentz force is increased when magnetic parameter is increased. Lorentz force creates retardation force into fluidic particles. So, this retardation force slows down flow regarding nanoparticles. Therefore, thickness regarding momentum layers is declined versus adjustment of magnetic parameter. But thickness of momentum layers is decreased when Lorentz force is enhanced. Further, thickness of momentum layers for $M=0$ is higher than thickness of momentum layer for $M\ne 0.$ Hence, tri-hybrid nanoparticles are observed as more efficient as compared to hybrid nanoparticles and nanofluid. The change in porosity number on velocity curves including impacts of tri-hybrid nanoparticles, nanoparticles and hybrid nanoparticles is captured by Fig. 5. Motion of nanoparticles is inclined due to retardation force created by porosity number. BLT (boundary layer thickness) associated with tri-hybrid nanoparticles are observed as more strong rather BLT associated with hybrid nanoparticles and nanoparticles. It is noticed that porosity number is created due to using porous surface in developed momentum equation. The term related to $(k_{1} f^{\prime})$ is negative term in momentum equation. So, opposite behaviour is investigated versus flow and porosity number. Therefore, acceleration becomes slow down when porosity number is increased. Figure 6 illustrates the impact of Parndtl number against motion of nanoparticles within nanoparticles. It is noticed that parameter related to $\alpha$ is modelled due to appearance of Prandtl fluid. The large values of Parndtl number make reduction in viscosity of nanoparticles. The appearance of $\alpha$ is produced using tensor regarding Prandtl fluid in momentum equation. The direct proportional relation is investigated among fluidic flow and $\alpha .$ Therefore, maximum acceleration is produced versus higher values of Prandtl number. Additionally, thickness regarding momentum layer is increased versus higher numerical values of $\alpha .$ Fluid becomes less viscous against variation in Prandtl number. Tri-hybrid nanoparticles and hybrid nanoparticles produce an enhancement in motion of fluid particles as compared motion is induced by nanoparticles. The visualization of elastic parameter versus the variation of heat energy is captured by Fig. 7. The reduction in the motion of nanoparticles is visualized when elastic number is increased. It is observed that velocity profile is decreased versus higher numerical values of elastic parameter. This kind of behaviour is investigated^5^ and viscosity is increased when elastic parameter is enhanced. Therefore, fluid becomes more viscous and motion into fluid particles is decreased. Physically, elastic nature is responsible for adjusting the thickness regarding viscous region. From this graph, less motion is created by nanoparticles as compared motion is produced by tri-hybrid nanoparticles and nanoparticles. Figure 8 is captured to estimate thermal features versus the variation in heat energy. It is estimated that two kinds of behaviour trends are noticed regarding heat absorption and heat generation. Velocity profile is enhanced when ${H}_{s}$ is increased. Because, external heat source is placed at wall of surface and thickness regarding layers of momentum can be controlled by change in heat source number.

### Comparative heat energy among nanoparticles via various parameters

**Figure 9:**
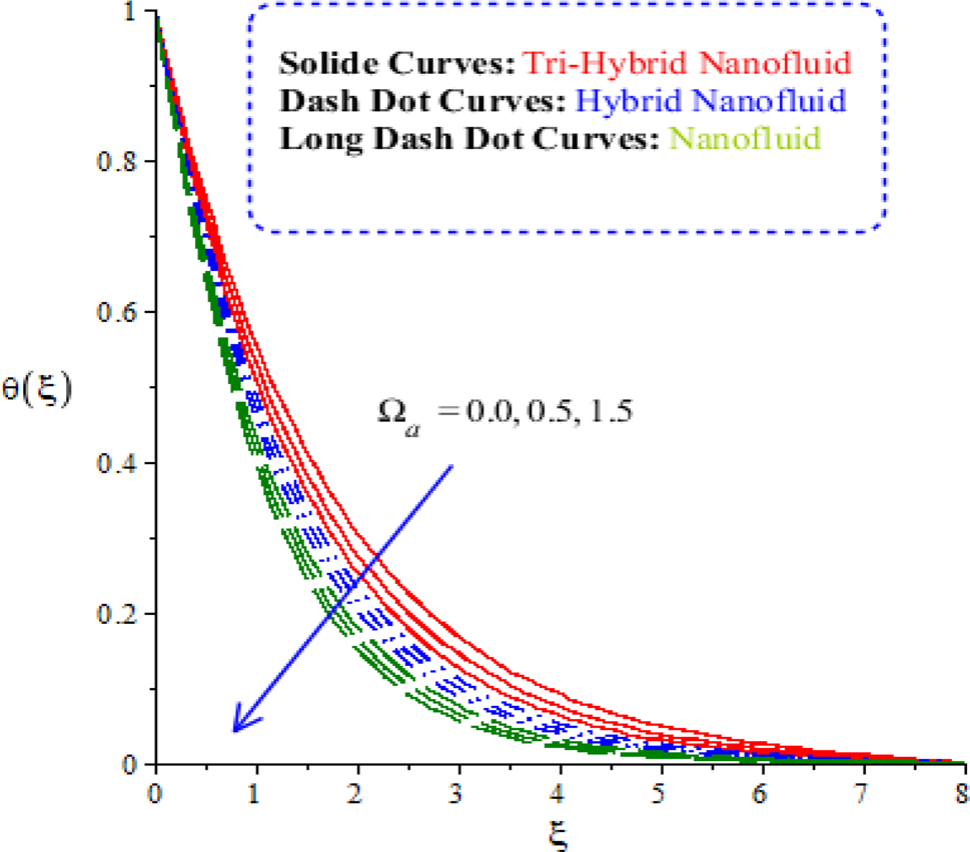
Comparative variation of temperature curves among nanoparticles versus ${\Omega }_{a}.$

**Figure 10:**
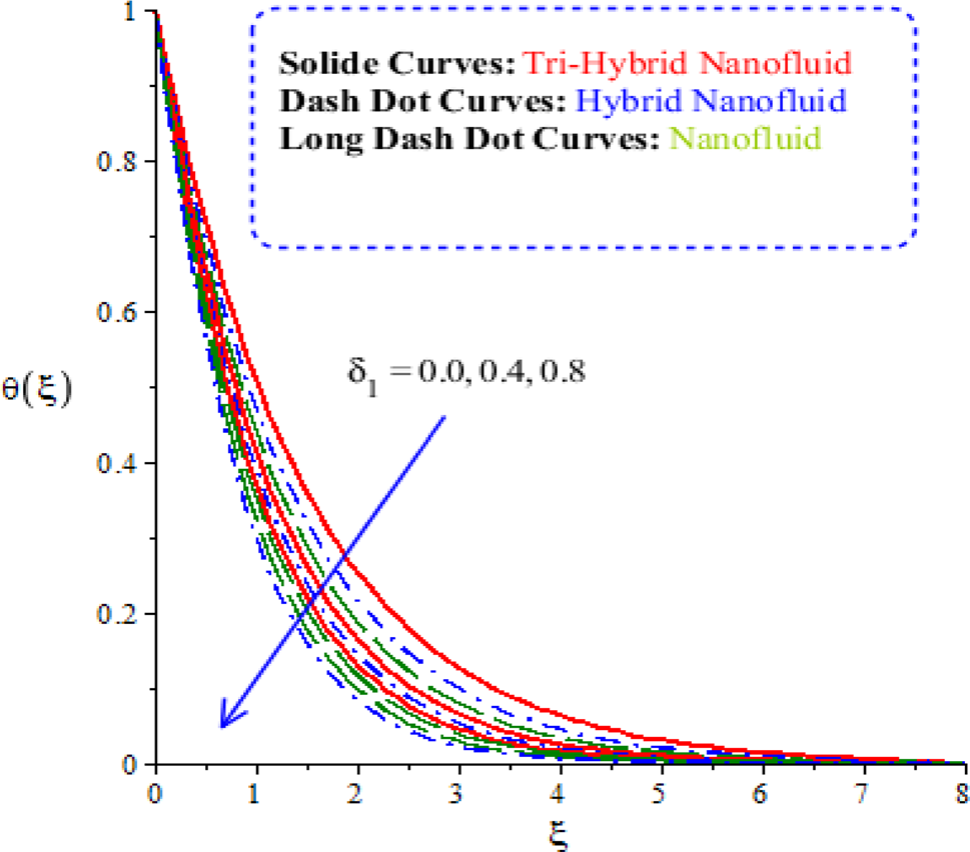
Comparative variation of temperature curves among nanoparticles versus ${\updelta }_{1}.$

**Figure 11:**
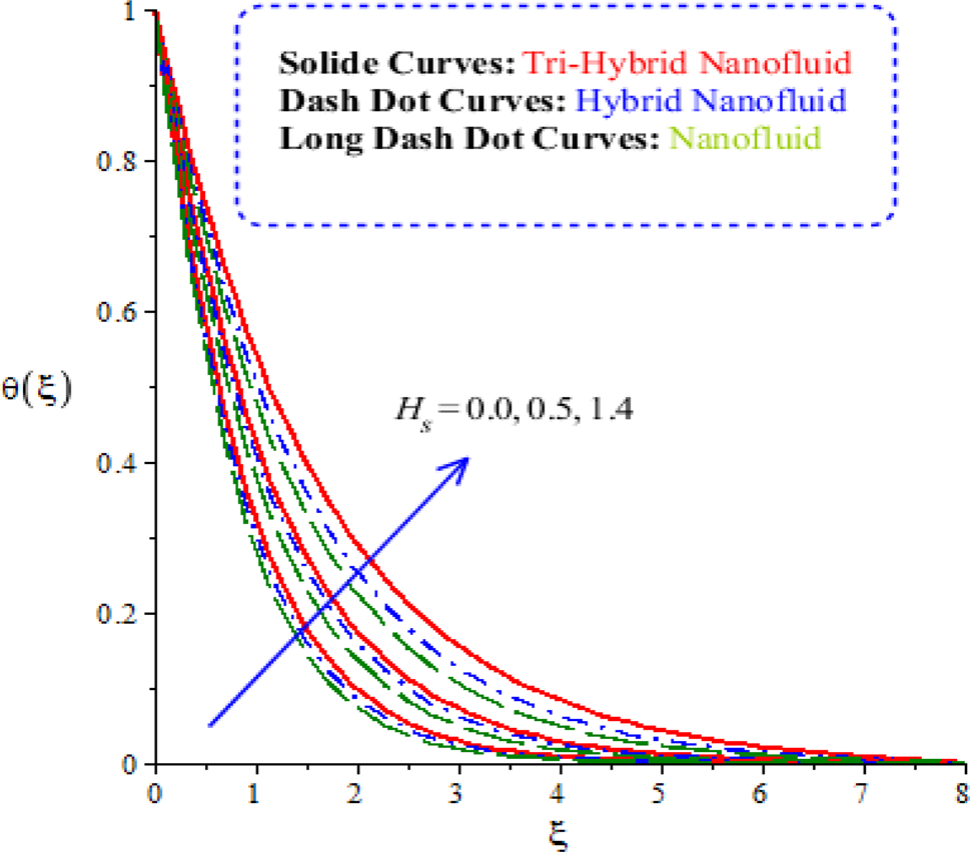
Comparative variation of temperature curves among nanoparticles versus ${H}_{s}.$

The features of heat energy along with hybrid nanoparticles, tri-hybrid nanoparticles and nanoparticles are investigated against variation of time relaxation, conductive and heat generation numbers. The features of heat energy and flow phenomenon are carried out by Figs. 9, 10 and 11. The role of time relaxation number against the variation of heat energy is captured by Fig. 9. The parameter related to ${\Omega }_{a}$ is formulated in energy equation because of non-Fourier’s law. The disappearance of ${\Omega }_{a}$ is revealed that appearance of Fourier’s law (classical case). It is estimated that higher heat energy is produced by Fourier’s law of heat conduction as compared by the case of non-Fourier’s law. The parameter regarding ${\Omega }_{a}$ reveals thermal relaxation number while an increment in thermal relaxation number results to make maximum amount regarding ability of fluidic particles to restore equilibrium condition is addressed. This impact creates minimizing variation in state regarding fluid. Thickness of thermal layers is decreased versus variation in thermal relaxation parameter. Moreover, production of heat energy is generated by tri-hybrid nanoparticles is estimated that more efficient as compared than heat energy is generated by hybrid nanoparticles and nanoparticles. Hence, heat energy becomes reduce against the variation of heat generation number. Distribution of heat energy is measured versus the variation of conductive number. The parameter related to ${\delta }_{1}$ is generated due to application of vertical melting surface. Heat energy is reduced versus the variation of ${\delta }_{1}$ as drafted through Fig. 10. TBLs (thermal boundary layers) associated with heat energy are decreased using higher values of conductive number. Moreover, TBLs along with tri-hybrid nanoparticles are higher than TBLs are generated by nanoparticles and hybrid nanoparticles. Mathematically, inverse proportional relation is predicted among heat energy and porosity number. Therefore, fluidic temperature is reduced when porosity parameter is increased. Further, porosity number is defined as $\left(\frac{{\nu }_{f}}{{aK}_{*}}\right)$. From definition of porosity number, it is investigated that porosity number has direct proportional relation against viscosity of fluidic particles. So, an increment in porosity number results viscosity of particles is increased. Therefore, flow becomes slow down. Figure 11 reveals behaviour of heat energy against the change in heat generation number. In this graph negative values of heat generation number are considered due to heat absorption while positive values of heat generation number are due to heat generation phenomena. Heat energy is increased because of external heat source. This external heat source makes the more production of heat energy by applying heat generation number. This is happened because heat energy adds into fluidic particles. Therefore, heat energy can be managed by the variation in heat source number. External heat source is implemented at surface. Therefore, heat energy is boosted. More production of heat energy is produced via tri-hybrid nanoparticles rather than heat energy is produced by hybrid nanoparticles and nanoparticles.

### Comparative surface force and temperature gradient among nanoparticles via various parameters

Distribution of surface force and temperature gradient against the variation of $M, {\delta }_{1}, {H}_{s}$ and $\alpha$ is simulated. A comparative study among nanoparticles is carried out by Table 4. It is noticed that ternary hybrid is observed as more significant to conduct maximize heat energy and motion in nanoparticles rather than for case that hybrid nanoparticles and nanoparticles. Ternary hybrid and hybrid nanoparticles are investigated that more significant process to bring maximizes motion and heat energy among nanoparticles. Maximum amount of temperature gradient is achieved versus the change in magnetic, elastic and heat generation number but a decline is investigated in rate of heat energy via applying large values of conductive number. The surface force is accelerated using large values of magnetic number whereas surface force at surface of wall is reduced using higher values of heat generation, elastic and conductive numbers. Related outcomes of variation of surface force and rate of heat transfer is simulated by Table 4.

**Table 4:** Comparative numerical values among nanoparticles of temperature gradient and surface force against change in $M, {\delta }_{1}, {H}_{s}$ and $\alpha$ via 300 elements.

		Nanoparticles		Hybrid nanoparticles		Tri-hybrid nanoparticles	
		${-\left(Re\right)}^{1/2}{C}_{f}$	${-\left(Re\right)}^{-1/2}Nu$	${-\left(Re\right)}^{1/2}{C}_{f}$	${-\left(Re\right)}^{-1/2}Nu$	${-\left(Re\right)}^{1/2}{C}_{f}$	${-\left(Re\right)}^{-1/2}Nu$
$M$	0.0	0.94548	0.37782	1.94285	1.38131	2.94281	2.18146
	0.3	0.93831	0.22007	1.95636	1.22471	2.93618	2.22509
	0.5	0.95345	0.19494	1.96401	1.10234	2.91401	2.30245
	0.0	0.75787	0.30266	1.75468	1.84040	2.75449	2.84141
${\delta }_{1}$	0.7	0.41858	0.59384	1.31591	1.91627	2.61633	2.91755
	1.3	0.34974	0.78940	1.14843	1.10588	2.34843	2.10639
	0.0	0.45877	0.27926	1.45382	1.28035	2.45554	2.28129
${H}_{s}$	0.5	0.31688	0.16086	1.31279	1.16081	2.31069	2.16035
	1.4	0.18325	0.04604	1.27758	1.04440	2.17582	2.12294
	0.0	0.62635	0.31687	1.62677	1.51801	2.62635	2.51774
$\alpha$	0.3	0.35013	0.26074	1.37555	1.44129	2.37555	2.34178
	1.3	0.27553	0.13038	1.10720	1.24458	2.10739	2.14510

## Prime findings and consequences

Thermal characteristics among tri-hybrid nanoparticles in rheology of Prandtl fluid over melting vertical surface are addressed. Non-Furrier’s law is used in energy equation along with heat generation phenomena. FEA (finite element approach) is utilized to know numerical as well as graphical outcome related velocity and temperature versus various parameters. The convergence analysis is confirmed via 300 elements. The main findings of current study are listed below:

- Fluidic motion for tri-hybrid nanoparticles is higher than fluidic motion for pure fluid, nanofluid and hybrid nanomaterial;
- Maximum thermal performance can be achieved for the case of tri-hybrid nanomaterial rather than fluid, nanofluid and hybrid nanomaterial;
- Present development is applicable in coolants regarding automobiles, dynamics in fuel and production of solar energy;
- Lorentz force reduces distribution into fluidic motion but opposite trend was investigated versus change fluid parameters;
- Non-Fourier’s approach declines thermal distribution and heat transfer rate is declined versus higher values of heat source number.

## Data Availability

The datasets generated/produced during and/or analyzed during the current study/research are available from the corresponding author on reasonable request.

## List of symbols

$\widetilde{{\varvec{\tau}}}$: Fluid vector tensor $\left({\mathrm{N}}\,{\text{m}}^{2}\right)$
$$V, U$$: Velocity components $$\left({\mathrm{m}}\,{\text{s}}^{-1}\right)$$
$$\nu$$: Kinematic viscosity $$\left({\mathrm{m}}^{2}\,{\mathrm{s}}^{-1}\right)$$
${\beta }_{1}$: Coefficient related to thermal exapnsion
$${T}_{\infty }$$: Ambient temperature $$(\mathrm{K})$$
$${k}_{*}$$: Prosity number
${\lambda }_{A}$: Time relaxation number
$$\rho$$: Fluid density $$\left({\mathrm{kg}}\,{\text{m}}^{-3}\right)$$
$${T}_{w}$$: Wall temperature $$(\mathrm{K})$$
$a$: Stretching number along x-axis $\left({\mathrm{m}}^{2/3}\,{\mathrm{s}}^{-1}\right)$
$$f$$: Dimensionless velocity
EG: Ethylene glycol
$$Pr$$: Prandtl number
FEA: Finite element approach
$$bf$$: Base fluid
${H}_{s}$: Heat source number
$${Q}_{w}$$: Heat flux
$${w}_{1}, {w}_{2}, {w}_{3}$$: Weight functions
$$\beta$$: Elastic number
$$A$$: Fluid number
$$x, y$$: Space coordinates $$(\mathrm{m})$$
$$G$$: Gravitational force $$(\mathrm{N})$$
$$T$$: Fluid temperature $$(\mathrm{K})$$
BCs: Boundary conditions
$Thnf$: Ternary hybrid nanofluid
$Q$: Heat source/absorption $(\mathrm{J})$
${C}_{p}$: Specific heat capacitance $\left({\mathrm{J}}\,{\text{kg}}^{-1}\mathrm{K}\right)$
$$\xi$$: Independent variable
$$\theta$$: Dimensionless temperature
$$\sigma$$: Electrical conductivity $$\left({\mathrm{S}}\,{\text{m}}^{-1}\right)$$
FEM: Finite element method
$${M}^{2}$$: Magnetic number
$${k}_{1}$$: Prosity parameter
${\Omega }_{a}$: Time relaxation number
$$Nu$$: Nusselt number
$$Re$$: Reynolds number
$$\infty$$: Infinity
ODEs: Ordinary differential equations
